# Heterogeneity in Multiple Sclerosis: Scratching the Surface of a Complex Disease

**DOI:** 10.4061/2011/932351

**Published:** 2010-12-09

**Authors:** Giulio Disanto, Antonio J. Berlanga, Adam E. Handel, Andrea E. Para, Amy M. Burrell, Anastasia Fries, Lahiru Handunnetthi, Gabriele C. De Luca, Julia M. Morahan

**Affiliations:** ^1^Wellcome Trust Centre for Human Genetics, University of Oxford, Roosevelt Drive, Headington, Oxford, OX3 7BN, UK; ^2^Department of Clinical Neurology, University of Oxford, The West Wing, John Radcliffe Hospital, Oxford, OX3 9DU, UK; ^3^Nuffield Department of Clinical Medicine, University of Oxford, Henry Wellcome Building for Molecular Physiology, Old Road Campus, Headington, Oxford OX3 7BN, UK

## Abstract

Multiple Sclerosis (MS) is the most common demyelinating disease of the central nervous system. Although the etiology and the pathogenesis of MS has been extensively investigated, no single pathway, reliable biomarker, diagnostic test, or specific treatment have yet been identified for all MS patients. One of the reasons behind this failure is likely to be the wide heterogeneity observed within the MS population. The clinical course of MS is highly variable and includes several subcategories and variants. Moreover, apart from the well-established association with the HLA-class II DRB1*15:01 allele, other genetic variants have been shown to vary significantly across different populations and individuals. Finally both pathological and immunological studies suggest that different pathways may be active in different MS patients. We conclude that these “MS subtypes” should still be considered as part of the same disease but hypothesize that spatiotemporal effects of genetic and environmental agents differentially influence MS course. These considerations are extremely relevant, as outcome prediction and personalised medicine represent the central aim of modern research.

## 1. Introduction

Multiple Sclerosis (MS) is a debilitating disease of the central nervous system (CNS) pathologically characterized by myelin loss and axonal degeneration. Although more than 100 years have passed since Charcot, Carswell, Cruveilhier, and others described the clinical and pathological characteristic of MS, both the etiology and the pathogenesis of this disease are not yet conclusively known [1]. 

With no reliable diagnostic test currently available, MS remains a clinical diagnosis with supportive paraclinical evidence. The basis of diagnosis is to clinically establish that disease activity has affected more than one part of the CNS and on more than one occasion (dissemination in time and space). This may be supplemented by investigations such as MRI, cerebrospinal fluid (CSF) electrophoresis, and evoked potential testing [1].

Both genetic and environmental factors have been shown to increase the risk of MS and only a few features are shared by most MS patients: the presence of inflammation, demyelination, and axonal loss within the CNS, a history of Epstein-Barr virus (EBV) infection and the detection of non-specific oligoclonal IgG bands in the CSF which have been shown in up to 95% of the MS patients [2, 3].

However, no common target antigen has been identified, no single diagnostic test is currently available and reliable biomarkers of disease activity are also lacking. Additionally, MS is characterized by a very broad and extensive heterogeneity in terms of clinical features, genetics, pathogenesis and responsiveness to treatments. Taken together, these observations have raised the question of whether MS is more a spectrum of diseases rather than a single entity. In this paper we aim to provide an updated analysis of the clinical, genetic, pathological, and immunological heterogeneity in MS.

## 2. Clinical Features

The differential diagnosis of MS is not straightforward. Several conditions such as infections, cerebrovascular diseases and autoimmune diseases can mimic the clinical features and the white matter changes seen in MS. Moreover, a few disorders are considered as MS variants and patients suffering from these conditions can either later develop a classic form of MS or show a disease course which is indistinguishable from that of classic MS. Thus, within the MS spectrum we can distinguish between classic MS (and its subcategories) and MS variants (Table 1) [4].

### 2.1. Classic MS

The clinical course of classic MS is highly variable, ranging from individuals showing occasional sensory nuisance to patients with fulminant course and death within months after disease onset. 

Approximately 85% of MS patients present with a clinically isolated syndrome (CIS) and later develop the relapsing-remitting form (RRMS), in which acute exacerbations are followed by periods of remission of symptoms. With time, recovery from each episode is incomplete and persistent symptoms accumulate. Approximately 70% to 80% of RRMS cases will enter the secondary progressive phase (SPMS) [1, 4]. About 15% of MS patients develop the primary progressive form of MS (PPMS), which is characterized by a gradually progressive clinical course from disease onset. Finally, a small group of patients are diagnosed with progressive relapsing MS (PRMS) in which only partial or no recovery occurs after exacerbations and disability accumulates in a stepwise manner. 

Further complicating this clinical scenario, the MS course is highly variable even within subgroups. The clinical outcome of RRMS cases varies from very mild forms of disease, wherein only minimal disability (Expanded Disability Status Scale, EDSS < 3) is attained over a period greater than 20 years from disease onset (mild MS) to rapidly progressive forms in which secondary progression is achieved in a few years (malignant MS) [5]. Moreover, during the secondary progressive phase of MS, disability progression can be acquired either because of a failure to recover from relapses (relapsing SPMS) or in the absence of clinically evident relapses (non relapsing SPMS) [6, 7]. Variability in disease outcome is also present in PPMS. In a recent study, the time to reach an EDSS of 6 was measured in a large cohort of PPMS patients. Interestingly, the rate of progression was shown to be slower than in other previous studies (14 years versus 7.1 years and 8.5 years to an EDSS of 6). Moreover, a marked variability was found within the same PPMS cohort with 25% of the patients reaching an EDSS of 6 in less than 7.8 years and another 25% in more than 27 years [8–10].

Poor outcome variables include male gender, frequent relapses in the first two years, a short period between the first and second attack, the absence of full recovery after the first attack, a high baseline T2 load on MRI, motor and cerebellar clinical signs, and African ethnicity [1, 4, 5, 11]. However, the reasons behind this variability are still unknown and although patients with benign disease for 10 years or longer tend to remain stable and not progress, the long-term clinical outcome of MS remains largely unpredictable [12].

### 2.2. MS Variants

Four conditions are known to closely resemble the classic form of MS and as yet it is not clear to what extent MS and its variants share common etiological and pathological features.

Neuromyelitis optica (NMO) or Devic's disease is a severe demyelinating disease of the CNS which preferentially affects the spinal cord and the optic nerve [13]. Although several epidemiological and clinical features discriminate between NMO and MS, whether these two conditions were two completely different entities or two faces of the same coin has long been debated. An important distinguishing finding was the detection in the serum of NMO patients of a specific antibody binding to aquaporin 4, a channel playing a central role in water homeostasis in the CNS [14]. The consequent detection of the same antibody in patients suffering from the Asian optical-spinal form of MS has led to the hypothesis that NMO and this particular form of MS may represent the same entity [13].

Marburg's variant of MS is characterized by fulminant demyelination and severe axonal loss which rapidly leads to extreme disability and sometimes death. A similar disease course is present in Balo's concentric sclerosis in which the pathological hallmark is the presence of lesions (detectable by MRI) characterized by concentric rings of demyelinated and normal tissue. Finally, Schilder's disease is a demyelinating disorder typically affecting children and characterized by large and confluent white matter lesions. Further details on MS variants can be found elsewhere [15, 16]. 

The presence of these variants and the fact that NMO is now acknowledged as a separate entity from MS raise the question as to whether analogous differences may be responsible for further stratification within the MS spectrum.

## 3. Genetics

### 3.1. Heterogeneity at Susceptibility Loci

A major role in determining genetic susceptibility to MS is played by the Human Leukocyte Antigen (HLA) genes which reside within the major histocompatibility complex (MHC) region. Each HLA allele is characterized by sets of digits separated by colons. The first set of digits describes the allele group, which often corresponds to the serological antigen. The second set of digits is used to distinguish alleles which are part of the same group but differ in the amino acid sequence of the encoded protein.

An association between MS and the MHC was demonstrated for the first time in the 1970s [17]. The association was later fine mapped to the extended class II haplotype HLA-DRB5*01:01-HLA-DRB1*15:01-HLA-DQA1*01:02-HLA-DQB1*06:02 in north Europeans [18] and it is now widely acknowledged that a predominant role is played by the HLA-DRB1*15:01 allele. Notably, this allele has been found to increase the risk of MS in nearly all the populations studied and an admixture scan of an African American cohort further suggested a major role for HLA-DRB1 [19–21]. 

On the other hand, several HLA-DRB1 alleles have been either positively or negatively associated with MS and these associations vary significantly across populations [22–29]. For example, in Sardinians MS is associated with the DRB1*03:01, DRB1*04:05 and DRB1*13:03 alleles [29]. Conversely, other allele groups such as DRB1*01, DRB1*10, DRB1*11 and DRB1*14 in Canadians and DRB1*09 in Japanese have been shown to exert a protective effect [26–28, 30]. Additionally, several studies have investigated the presence of HLA-class I alleles acting independently of class II loci. HLA-A*02, HLA-B*44 and HLA-Cw*05 alleles have been shown to decrease the risk of MS after conditioning on the presence of DRB1*15:01 [31–34]. A current list of HLA-class I and class II MS-associated alleles is provided in Table 2. 

**Figure 1:**
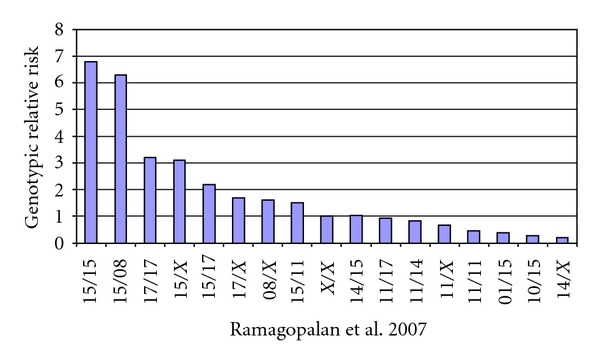
The relative risk of MS is determined by *trans epistasis* between different HLA-DRB1 alleles.

This scenario is further complicated by the extensive linkage disequilibrium of the MHC region and the presence of *cis* and *trans epistasis* between different HLA-class II genes (Figure 1) [26, 27, 35, 36].

However, the MHC is not the only a genetic region associated with MS susceptibility. Recent genome wide association (GWA) studies revealed the existence of multiple non-MHC MS susceptibility loci of modest effect [37–54]. A current list of the well-established associated variants is shown in Table 3. 

The vast majority of these genes are involved in the immune system, and this supports the hypothesis that MS is an immune-mediated disorder of the CNS. However, as evidenced by their wide expression profile (see Table 3), different pathways in both the innate and adaptive immune responses are likely to be involved in MS pathogenesis. Intriguingly, another MS-associated gene (*KIF1B*) encodes a kinesin superfamily member which is believed to be responsible for axonal transport of mitochondria and synaptic vesicles precursors, suggesting that also a primary neurodegenerative component may play a role in MS [47].

In addition to these genes, several others have been associated with MS but currently lack replication. However, this does not necessarily mean false positive association. A careful ascertainment of cases and controls is a fundamental requirement which is not easily achieved, especially in a heterogeneous disease such as MS. Moreover, even in a perfectly designed study, the lack of replication could be simply explained by a diverse role played by the same variant in different populations. Genes such as *STAT3* and *CBLB* have been associated with MS in the Finnish and Sardinian MS populations respectively, but have not been replicated by other studies. Interestingly, STAT3 is a transcription factor involved in the differentiation of naïve CD4+ T cells into Th17 cells, while CBLB has been shown to negatively regulate both T and B cell receptor activations [55, 56]. Although a false positive association may well be responsible for this inconsistency, the immunological role played by these genes raises the hypothesis that some genetic variants may be either more easily identified or etiologically more relevant in certain isolated populations.

### 3.2. Heterogeneity at Outcome Loci

Several studies have also investigated the association between genetic variants and clinical outcome. In a Canadian report, the HLA-DRB1 allele frequencies were compared between mild (RRMS with EDSS ≤ 3 over a period >20 years) and malignant (PPMS or RPMS with EDSS > 6 within 5 years of disease onset) MS cases. DRB1*01 was shown to be protective against a severe disease course in both sporadic and familial MS. Intriguingly, in the familial cases the protective effect of DRB1*01 was only significant when it was part of the DRB1*01-DRB1*15:01 genotype. HLA-DRB1*15:01 was instead equally distributed between mild and malignant MS patients, although a greater proportion of DRB1*15:01 homozygous patients was found in the malignant group [57]. A protective role for DRB1*01 was then confirmed in an Australian cohort of 984 RRMS and 246 PPMS patients, but only in the presence of DRB1*15 on the other allele (similarly to the Canadian familial cases). Additionally, DRB1*04 was also negatively associated with PPMS [58]. 

Conversely, in a Spanish MS cohort, both DRB1*01 and DRB1*04 were found to be associated with a shorter time to reach an EDSS of 6 [59]. Finally, in a large French study, a positive correlation between DRB1*15:01 and disease progression was shown in the RRMS but not in the PPMS groups [60]. While these findings seem conflicting it may be due to differences in study design: comparing PPMS with RRMS may fail to elicit important outcome effects given the tremendous clinical variability within the MS subgroups. Also, as mentioned previously, the same variant may play diverse roles in different populations. 

HLA genes are thought to be involved in immune-mediated diseases through their role in antigen presentation. Thus one reason different HLA-DRB1 alleles may lead to different outcomes among MS patients may be due to antigen specificity. The myelin sheath is a complex structure comprised of various types of lipids (glycosphingolipids, cholesterol, and phospholipids) and proteins including proteolipid protein (PLP), myelin basic protein (MBP), myelin-associated glycoprotein (MAG), myelin-oligodendrocyte glycoprotein (MOG), and 2′ 3′-cyclic-nucleotide-3′phosphodiesterase (CNP) [61]. All of the above components have been suggested as candidate antigens, but to date there is no verified antigen for MS [61]. The complexity of the disease together with the heterogeneity of the MHC associated alleles would suggest that the different myelin components or the entire complex structure of the myelin sheath may be the target of the immune reaction. Differences in antigen specificity and the role played by the protein within the myelin sheath may lead to differences in clinical outcome in a patient-specific manner. 

Non-MHC loci have also been investigated and a number of genes have been associated with different markers of disease phenotype such as age of onset, disease severity, lesion load and brain atrophy. Interestingly, a gene-ontology analysis showed that many of these genes were involved in neural processes and several cellular mechanisms, but further studies are needed to confirm these findings [62].

## 4. Pathology

### 4.1. Relapsing versus Progressive MS

The pathological hallmark of MS is the sclerotic plaque, which represents the end stage of a process involving inflammation, demyelination, remyelination, astrocytosis, and axonal degeneration. However, the order in which these processes take place is still unknown [1].

In the relapsing-remitting phase, the classical pathological finding is active white matter plaques in which inflammatory demyelination clearly plays a central role. Myelin-laden macrophages and (to a lesser extent) CD8+ T cells dominate the lesions, while CD4+ T cells (both Th1 and Th17) are found primarily in the perivascular regions and with relatively smaller numbers in the parenchyma [63–66]. Cortical demyelinating lesions are also present and have been shown to correlate with cortical atrophy, disease progression, physical disability, and cognitive impairment at later stages [67–70]. Interestingly, cortical demyelination seems to be present since the relapsing-remitting phase but becomes more prominent during the secondary progressive phase [71]. Moreover, in contrast with those of the white matter, grey matter lesions typically show a very low grade of both T and B inflammatory infiltrates [67].

In the progressive phase of MS (both PPMS and SPMS), neurodegeneration proves the main pathological finding and occurs on the background of a compartmentalized pathological immune reaction which seems to act independently from the central immune system [64, 71]. T cells are still the main cell population found within chronic lesions but they are sparse and mainly located in perivascular spaces, while microglia, B cells, and plasma cells become increasingly prominent [72, 73]. Additionally, some studies have shown the presence of clusters of B cells resembling the structure of germinal centers inside the meninges [74, 75]. These B cells have been reported to bear EBV, although this finding lacks replication [76, 77]. Finally, inflammatory infiltrates are also detected in the normal appearing white matter (NAWM) in which T cells (mainly CD8+) and profound microglia activation are associated with diffuse axonal injury and do not correlate with the number, size, location, and destructiveness of active lesions [64, 71, 78].

### 4.2. Pathological Heterogeneity

The presence of heterogeneity in active white matter lesions has been largely debated since Lucchinetti et al. defined four distinct types of active plaques from a number of autopsy (*n* = 32) and biopsy (*n* = 51) samples, strongly suggesting a multiple disease hypothesis (Table 4) [7].

However, these findings must be interpreted with caution for several reasons: (1) Biopsy data are bound to be less representative and reliable than autopsy material [79]. (2) The pathological criteria used to define the activity of the plaques still lack a confident validation and this is likely to undermine the entire classification. (3) Complement activation (pattern II) is not easy to interpret in formalin-fixed tissue and has been shown to be an invariable and nonspecific feature of not only MS but also other white matter conditions [79–81]. (4) Apoptotic oligodendrocytes (pattern III) could be either mistaken for other apoptotic cells, in particular lymphocytes, or merely be the consequence of confounding factors such terminal hypoxia [79]. (5) Partial Balo lesions (pattern III) are a common finding in relapsing remitting patients and have been shown also in other patterns of MS lesions [81, 82]. (6) Finally, it is not clear to what extent these pathological findings should be seen in the lesions in order to confidently define them as part of a specific pattern. 

Taken together, these observations suggest that these different types of white matter lesions are more likely to be part of the same spectrum or reflect different stages of demyelination rather than representing single and distinct pathological entities [63].

It is now widely acknowledged that disease progression depends on accumulated neuronal degeneration and cortical atrophy. Whether these are reached as a consequence of inflammation and demyelination or represent an independent neurodegenerative process has long been debated. Theoretically, five pathways may be involved and responsible for neuronal damage: (1) white matter demyelinating lesions, (2) grey matter demyelinating lesions of which four different types have been described (Table 5) [83], (3) diffuse inflammation of the NAWM, (4) B cell follicles located in the meninges which have been shown to correlate with areas of cortical atrophy [64, 74], and (5) a primary independent neurodegenerative process [84, 85]. 

Rather than acting independently, these mechanisms are likely to act together but to a different extent in a patient specific manner. These differences would then lead to the pathological heterogeneity seen in MS.

## 5. Immunological Phenotype

### 5.1. Cell Type Complexity

For a long time, MS has been generally considered as a CD4+ T helper cell-(Th-) mediated immune disorder. This concept primarily arose from the HLA-class II association with MS susceptibility and from the central role played by Th cells in experimental autoimmune encephalomyelitis (EAE), the rodent model of MS, in which an MS-like demyelinating disease is induced by the injection of myelin-specific CD4+ T cells [86]. However, while the treatment with an antibody against the p40 subunit of IL-12, which is important for Th1 cell differentiation, could prevent EAE [87], the use of ustekinumab (another antibody for the same subunit) produced no benefit in Phase II clinical trials [88]. These results highlight the much greater complexity of MS immunopathogenesis when compared to the EAE model.

Interestingly, the most consistent immunological feature in MS is the presence of IgG oligoclonal bands which are detected in the CSF of up to 95% of the MS patients [3]. Although their specificity remains to be resolved, their presence stands for an abnormal B cell activation within the CNS. Other recent studies suggest a relevant role played by B cells in MS pathogenesis in terms of T cell activation, CIS conversion to MS, and development of disease progression [76, 77, 89–91]. The central role played by B cells in MS is further supported by the significant reduction of inflammatory lesions and clinical relapses observed when B cells are depleted using the anti-CD20 monoclonal antibody Rituximab [92, 93].

T cells are also important and several recent studies were aimed at the identification of the T cell subtypes primarily involved in the immunopathogenesis of MS. 

CD8+ T cells represent the largest T cell subset both in acute and chronic MS lesions. Moreover, they show oligoclonal expansion within the CNS strongly suggesting their contribution to MS pathogenesis [94–97]. 

Interleukin 17 (IL-17) producing T helper cells (Th17 cells) have been recently identified as a distinct subset of T cells strongly involved in autoimmunity [98, 99]. A central role for Th17 cells in MS has been suggested by several studies reporting: (1) the presence of IL-17+ T cells in active MS lesions [66], (2) an increased ability of CD4+ T cells taken from MS patients to produce IL-17 upon polyclonal mitogen or myelin-specific antigen stimulation [100], (3) higher frequency of Th17 in the CSF of CIS and RRMS patients in the relapsing rather than remitting phase [101], (4) higher expression of the transcription factor STAT 3 (which regulates the differentiation of CD4+ T cells into Th17 cells) during the relapsing phase of MS [102], and (5) the upregulation of miR-326 (a positive regulator of Th17 differentiation) in RRMS patients experiencing a relapse in comparison with remitting cases and healthy controls [103]. However, although these findings strongly support a role for Th17 cells in MS, whether these cells are causative or merely a marker of disease activity remains a challenging question.

The role of the main type of regulatory T cells (CD4+ CD25+ FOXP3+ Treg) in MS has also been extensively investigated. In RRMS patients, these Tregs display an impaired capacity to suppress both polyclonally activated and myelin-specific T cells as compared with controls [104–106]. Interestingly, a correlation between their suppressive function and vitamin D levels has also been reported giving a potential explanation for the association between vitamin D levels and relapse rate [107, 108]. Additionally, recent thymic emigrating Tregs seem to play a major role as they were shown to be reduced and to contain a significantly lower number of T cell receptor excision circles in RRMS as compared to normal controls [109, 110]. Finally, it must be noted that the CD4+ CD25+ FOXP3+ Tregs only represent one regulatory cell type and that other subsets have also been shown to be involved in MS. Further details on regulatory T cells in MS can be found elsewhere [111, 112]. 

These studies confirm the presence of a great immunological heterogeneity in the MS immune system with several different cell types all likely to be involved. Moreover, it has to be emphasized that in all the studies mentioned, differences between cases and controls are often very subtle and no immunological finding can at present be used as biomarkers of disease activity.

### 5.2. Individual Complexity

Most of the data for immunological phenotyping derives from studies performed in a limited number of patients, usually those with RRMS. However, even in these limited sets, heterogeneity can be appreciated. A recent study extensively investigated the cytometric profile of a large cohort of RRMS and CIS patients. Interestingly, both RRMS and CIS cases showed a decreased frequency of CD8^low^ CD56+ CD3− CD4− cells which have a natural killer (NK) profile, adding to the hypothesis that NK regulatory properties may also be reduced in MS [113]. Moreover, in the same study, both RRMS and CIS patients were shown to cluster into three distinct groups: the first was characterized by the lower frequency of CD8^low^ CD56+ CD3− CD4− cells while the second and third by changes in the frequencies of large granular and CD14+ cells, respectively [114]. 

Another recent study using EAE and RRMS patients showed differential response to interferon beta (IFN-*β*) treatment. Interestingly, IFN-*β* was more effective in Th1 as compared to Th17-induced EAE. Similarly, in RRMS patients a higher IL-17F concentration in serum was found in nonresponders as compared to responders. Non-responders also showed worse disease with steroid administration and had a higher number of relapses [115].

Finally, when considering sources of immunological variation in MS, it is interesting to note that the differentiation of Th17 and CD4+ CD25+ FOXP3+ Treg cells are tightly related. The differentiation of CD4+ naive T cells into Th17 cells or Tregs has been shown to be dependent on TGF *β* stimulation during antigen presentation. High levels of TGF *β* promote Treg production, while a low dose of TGF *β* exerts the opposite effect by increasing the expression of the Th17 transcription factor ROR*γτ* leading to the production of Th17 cells. The flexibility of the Th17-Treg system is further confirmed by the capacity of TGF *β* and IL6 to actually reprogram Tregs into Th17 cells through the ROR*γτ* and STAT3 pathways, respectively [111, 116]. Therefore, an immune system that was preferentially skewed towards the production of Th17 or Treg subsets may represent a further source of interindividual heterogeneity in MS and lead to a more or less severe relapse rate and clinical course.

Taken together, these studies strongly suggest that different cell types are likely to be involved in a patient-specific manner and that these differences are able to influence disease course and response to treatments.

## 6. Conclusions and Perspectives

We have seen how MS clinical features, genetics, pathology, and immunological phenotype show a high degree of variability between individuals and ethnicities. Notably, no single pathway, reliable biomarker, diagnostic test, and specific treatment have yet been identified for all MS patients. However, there are several commonalities among the MS subtypes: the association of HLA-DRB1*15:01 allele has been shown across wide variety of populations and within clinical subtypes of MS [20]; similarly, low vitamin D level is now an established environmental MS risk factor [108]; furthermore, it is striking that more than 99% of the MS patients have been found to have been infected with EBV [117]. These observations lead us to conclude that despite the wide heterogeneity, there is insufficient evidence to maintain that MS represents a spectrum of etiologically different disorders. We believe that genetic and environmental factors play a central role not only in triggering the onset but also in modifying the course of the disease by influencing individual neurological susceptibility and immunological responses. This is likely to lead to the wide clinical, pathological, and immunological heterogeneity observed in MS patients. 

The differences described in this review remain important considerations for accurate study designs as well as the ultimate goal of personalised treatments for MS patients. At present, the response to the currently approved therapeutic agents (IFN *β*, glatiramer acetate, mitoxantrone and natalizumab) varies significantly across the MS population. Moreover, no treatment is able to halt disease progression [118]. A clearer understanding of the heterogeneity within the MS phenotype is required in order to achieve effective treatment for all patients with MS.

## Figures and Tables

**Table 1:** Classic MS and its variants.

Classic MS	MS variants
(i) Relapsing-remitting (RRMS): 85% of all MS cases at onset	(i) Neuromyelitis Optica (NMO)
(ii) Second ary progressive (SPMS): 70%–80% of RRMS cases after 10 years from disease onset	(ii) Balo's concentric sclerosis
(iii) Primary progressive (PPMS): 15% of all MS cases at onset	(iii) Margburg's MS variant
(iv) Progressive-relapsing (PRMS): very small percentage	(iv) Schilder's MS variant

**Table 2:** Reported HLA class II and class I associations across the world.

	Population	Approximate OR	Reference
HLA-DRB1 alleles			
	Canada		[26, 27]
*01	Sweden	0.6	[32]
	UK, US		[31]
*03	Canada	1.7	[26, 27]
	Sweden,		[24]
	UK, US, Italy, Spain		[25]
	Sardinia		[29]
*04	Sardinia	2.2	[29]
*07	Italy	0.6	[22]
*08	Canada	1.7	[26, 27]
	UK, US, Italy, Spain	(15/8 genotype)	[25]
*09	Japan	0.4	[28]
*10	Canada	0.7	[26, 27]
*11	Canada	0.7	[26, 27]
*13	Sardinia	2	[29]
	Israel		[23]
*14	Canada,	0.3	[26, 27]
	UK, US, Italy, Spain		[25]
*15	Near-universal	3	

HLA-class I alleles			
A*02	Sweden	0.6	[33]
	Italy		[31]
B*44	UK, US	0.4	[34]
Cw*05	UK, US	<1	[32]

**Table 3:** List of established non-MHC MS-associated genes.

Gene	Proposed function	CH	OR	UCSC Microarray expression data	References
IL7Ra	Cytokine receptor	5	1.18	CD4+ T cells ++++, CD8+ T cells ++++,	[38–42, 44]
Interleukin 7 receptor				CD56+ NK +++, BCDA4+DCs ++,	
				CD14+ Monocytes+	
IL2Ra	Cytokine receptor	10	1.19	CD4+ T cells ++, CD8+ T cells +,	[37, 38, 40, 42]
Interleukin 2 receptor				CD56+ NK +	
CLEC16A	Sugar binding C type lectin	16	1.18	CD19+ B cells +, CD56+ NK +,	[45, 48, 49, 53]
C lectin domain A				BCDA4+DCs +	
CD58	Ligand of CD2/T cell activation	1	1.30	CD56+ NK ++++, CD14+ Monocytes++++,	[37, 38, 43, 45, 49]
				CD8+ T cells +++, CD19+ B cells++,	
				CD4+ T cells ++, BCDA4+DCs ++	
CD6	Cell signaling/T cell activation	11	1.18	CD4+ T cells ++++, CD8+ T cells ++++,	[54]
				CD56+ NK +++, BCDA4+DCs +	
IRF8	Interferon regulatory factor	16	0.80	CD19+ B cells ++++, BCDA4+DCs ++++,	[54]
Interferon regulatory				CD56+ NK ++, CD14+ Monocytes ++,	
factor 8				CD4+ T cells +, CD8+ T cells +	
CD226	Cell-cell adhesion	18	1.11	CD56+ NK ++	[50, 53]
TNFRSF1A	Tumor necrosis factor receptor	12	1.20	CD14+ Monocytes +++, CD56+ NK ++,	[54]
Tumor necrosis factor				BCDA4+DCs +, CD4+ T cells +,	
receptor 1				CD8+ T cells +	
EVI5	Cell cycle regulation	1	1.1	BCDA4+DCs +, CD14+ Monocytes +,	[37, 45, 51]
Ecotropic viral				CD19+ B cells+	
integration site 5					
CD40	Tumor Necrosis Factor receptor	20	1.20	CD56+ NK +, CD14+ Monocytes +,	[45]
	Super family member 5			BCDA4+DCs +	
TYK2	Cell signaling	19	1.32	CD56+ NK +++, CD14+ Monocytes +++,	[44, 45]
Tyrosine kinase 2				BCDA4+DCs +++, CD8+ T cells ++,	
				CD19+ B cells ++, CD4+ T cells ++	
KIF1B	Axonal transport	1	1.34	Whole brain ++++	[47]
Kinesin family member					
1B					

^+^Increasing number of crosses correspond to increasing expression levels.

**Table 4:** Patterns of demyelination described by Lucchinettiet al. 2000 [7].

Pattern of white matter demyelination	Pathology
(i) Macrophage mediated	(i) Perivenous distribution of lesions
	(ii) T cell and macrophage infiltrates
	(iii) Shadow plaques (remyelination)
	(iv) Sharp lesion edges

(ii) Antibody mediated	(i) As pattern I lesions
	(ii) Deposition of immunoglobulin and activated complement

(iii) Distal oligodendrogliopathy	(i) Important oligodendrocyte apoptosis
	(ii) T cell, macrophage, and microglia infiltrates
	(iii) Degeneration of distal oligodendrocyte processes
	(iv) Ill defined lesion edges
	(v) Preferential loss of myelin associated glyco-protein (MAG)
	(vi) Concentric Balo-like lesions

(iv) Primary oligodendrocyte damage	(i) Similar to pattern I
	(ii) Massive oligodendrocyte loss

**Table 5:** Types of cortical lesions described by B*∅* et al. 2003 [83].

Typeof cortical lesion	Extension
Type I	Extension through both white and gray matter
Type II	Lesion delimited within the cortex. Neither the brain surface nor the subcortical white matter is involved
Type III	Extended subpial lesions
Type IV	Extension throughout the full width of cerebral cortex but white matter is not involved
